# The Addition of Liquid Fructose to a Western-Type Diet in LDL-R^−/−^ Mice Induces Liver Inflammation and Fibrogenesis Markers without Disrupting Insulin Receptor Signalling after an Insulin Challenge

**DOI:** 10.3390/nu9030278

**Published:** 2017-03-15

**Authors:** Gemma Sangüesa, Miguel Baena, Natalia Hutter, José Carlos Montañés, Rosa María Sánchez, Núria Roglans, Juan Carlos Laguna, Marta Alegret

**Affiliations:** 1 Department of Pharmacology, Toxicology and Therapeutic Chemistry, School of Pharmacy and Food Sciences, University of Barcelona, 08028 Barcelona, Spain; gemmasanguesa@gmail.com (G.S.); miguelbiologo@gmail.com (M.B.); nataliahutter@yahoo.es (N.H.); josemontanyesbq@gmail.com (J.C.M.); rmsanchez@ub.edu (R.M.S.); roglans@ub.edu (N.R.); 2 Institute of Biomedicine, University of Barcelona, 08028 Barcelona, Spain; 3 CIBER Fisiología de la Obesidad y Nutrición (CIBEROBN), Instituto de Salud Carlos III (ISCIII), Spain

**Keywords:** simple sugars, high-fat diet, adipose tissue, inflammasome, metabolism

## Abstract

A high consumption of fat and simple sugars, especially fructose, has been related to the development of insulin resistance, but the mechanisms involved in the effects of these nutrients are not fully understood. This study investigates the effects of a Western-type diet and liquid fructose supplementation, alone and combined, on insulin signalling and inflammation in low-density lipoprotein (LDL) receptor-deficient mice (LDL-R^−/−^). LDL-R^−/−^ mice were fed chow or Western diet ±15% fructose solution for 12 weeks. Plasma glucose and insulin, and the expression of genes related to inflammation in the liver and visceral white adipose tissue (vWAT), were analysed. V-akt murine thymoma viral oncogene homolog-2 (Akt) activation was measured in the liver of the mice after a single injection of saline or insulin. None of the dietary interventions caused inflammation in vWAT, whereas the Western diet induced hepatic inflammation, which was further enhanced by liquid fructose, leading also to a significant increase in fibrogenesis markers. However, there was no change in plasma glucose or insulin, or insulin-induced Akt phosphorylation. In conclusion, hepatic inflammation and fibrogenesis markers induced by a Western diet supplemented with liquid fructose in LDL-R^−/−^ mice are not associated with a significant impairment of hepatic insulin signalling.

## 1. Introduction

Unhealthy nutritional habits such as the excessive consumption of fat and refined sugars, together with a sedentary way of life, are linked to metabolic alterations including non-alcoholic fatty liver disease (NAFLD)—an array of conditions ranging from simple steatosis to steatohepatitis. NAFLD is strongly associated with insulin resistance (IR). However, the role of hepatic lipid accumulation and inflammation in the alterations of insulin signalling which will lead to IR is not fully understood. For example, although in animal models hepatic steatosis has been generally linked to the development of IR, this association is not so straightforward in the studies in which adiposity and body weight are not affected [1]. On the other hand, inflammation has been proposed as the link between NAFLD and IR, as several murine models in which components of the nuclear factor κ B (NFκB) inflammatory pathway are constitutively activated or deleted show that increased hepatic inflammation correlates with an increase in IR [2] and, conversely, a reduction in hepatic inflammation improves hepatic insulin sensitivity [3,4,5]. However, the evidence is not so clear, as other murine models show a dissociation between inflammation in the liver and IR [6,7,8,9,10,11]. For example, mice with a gain-of-function mutation in tumour necrosis factor receptor 1 display hepatic inflammation, but systemic and hepatic IR are not affected and adipose tissue inflammation is not observed [10].

The food constituents that have been linked to the development of metabolic disturbances such as NAFLD and IR include simple sugars, especially fructose. Our research group has shown that liquid fructose supplementation in female rats fed a standard chow diet (two weeks to two months) induces glucose intolerance and IR [12,13,14,15]. However, unhealthy human dietary patterns frequently include not only excessive added sugars, but also excessive fat intake, particularly saturated fats [16]. For this reason, in a recent publication we explored the effects of liquid fructose supplementation to C57BL/6N mice in two different solid diets, a standard rodent chow and a Western-type rodent chow rich in saturated fat, refined carbohydrates and cholesterol [17]. Our results showed that in this model liquid fructose supplementation increased liver triglyceride and cholesterol levels and diminished whole-body insulin sensitivity only when it was combined with a Western-type diet; however, these effects were not associated with a clear inflammatory reaction in the liver [17]. We also studied the effects of these diets on the development of aortic atherosclerotic lesions in low-density lipoprotein (LDL) receptor-deficient (LDL-R^−/−^) mice [18]. As it has been shown that the LDL-R^−/−^ mouse is a good model for studying the progression from steatosis to inflammation in the liver [19], it can be helpful to better understand the relationship between hepatic lipid accumulation, inflammation and IR. Here, we investigate the effects on insulin signalling and inflammation of liquid fructose supplementation and a Western-type diet, alone and combined, using samples from our previous study conducted on LDL-R^−/−^ mice [18].

## 2. Materials and Methods

### 2.1. Animals and Diets

The animal experimental protocols were described in our previous publication [18]. Briefly, male LDL-R^−/−^ mice were randomly assigned to four groups (*n* = 10/group): 1. Control group (CT), which was fed a standard rodent diet (2018 Teklad Global 18% Protein, Envigo, Barcelona, Spain) without any supplementary sugar; 2. Fructose-supplemented group (F), which received the same standard diet supplemented with 15% weight/volume fructose in drinking water; 3. Western group (W), which was fed a Western-type diet (D12079B Open Source Diets, Research Diets Inc., New Brunswick, NJ, USA) without any supplementary sugar; 4. Western plus fructose group (WF), which received the Western-type diet supplemented with 15% weight/volume fructose in drinking water. The composition of control and Western-type diets was as detailed previously [18]. After 12 weeks, the animals were fasted for 2 h and blood was obtained from the tail vein to determine glucose concentrations. Six animals from each group were then euthanized under intraperitoneal ketamine (100 mg/kg)/xylazine (10 mg/Kg) anaesthesia. Blood samples were obtained by intracardiac puncture, collected in microtubes (Sarstedt AG & Co., Nümbrecht, Germany), and plasma was obtained by centrifugation and stored at −80 °C until use. Liver and visceral white adipose tissue (vWAT) was excised, immediately frozen in liquid N_2_ and stored at −80 °C until use. The remaining animals (*n* = 4/group) were used to study insulin signalling in the liver. To this end, mice were fasted for 12 h, anaesthetised with ketamine:xylazine, as described above, and intraperitoneally injected with 0.15 units of insulin/g body weight (Humulina^®^ Regular, Lilly, Madrid, Spain). Fifteen minutes later the livers were obtained, frozen in liquid N_2_ and stored at −80 °C.

All procedures were conducted in accordance with the guidelines established by the University of Barcelona’s Bioethics Committee, as stated in Law 5/1995, of 21 July, from the Catalan government (approval code 6078). These guidelines adhere to Directive 2010/63/EU of the European Parliament on the protection of animals used for scientific purposes.

### 2.2. Blood and Plasma Analysis

Blood glucose levels were measured using an Accutrend^®^ Plus system glucometer (Cobas, Roche Farma, Barcelona, Spain). Insulin concentration in plasma was determined using an ELISA kit (Millipore, Billerica, MA, USA). Whole-body insulin sensitivity was estimated by calculating the Insulin Sensitivity Index (ISI) [20], and the HOMA-IR [21]. Alanine aminotransferase (ALT) activity in plasma samples was determined using an ALT/GPT Spinreact kit (Spinreact, Girona, Spain), based on the spectrophotometric measurement of the rate of reduction in NADH levels. To determine lipopolysaccharide-binding protein (LBP) levels, we used an ELISA kit from Hycult Biotech (Uden, The Netherlands).

### 2.3. Histological Studies

Paraffin-embedded liver sections were stained with hematoxylin and eosin to assess necrosis and with Masson’s trichrome acid to determine the degree of fibrosis. Images were acquired with an Olympus BX43 microscope (Olympus Iberia, Barcelona, Spain) and a pathologist blinded to the treatment groups performed the histological analysis at BioBanc (Banc de tumors-IDIBAPS, Barcelona, Spain). Necrosis was scored as 0 (absent), 1 (<1%), 2 (<5%), 3 (<10%) or 4 (≥10%).

Fibrosis was categorised as 0 (no fibrosis), 1 (portal or sinusoidal fibrosis without septa), 2 (portal or sinusoidal fibrosis with rare septa), 3 (abundant septa without cirrhosis) or 4 (cirrhosis).

### 2.4. RNA Preparation and Analysis

Total RNA was isolated by using Trizol^®^ Reagent (Invitrogen, Carlsbad, CA, USA), in accordance with the manufacturer’s instructions. Specific mRNAs were assessed by real-time reverse transcription polymerase chain reaction (RT-PCR), using SYBR Green PCR Master Mix, specific primers and the Applied Biosystems StepOnePlus sequence detection system (Applied Biosystems, Foster City, CA, USA). TATA box-binding protein (TBP) was used as an internal control. Primer sequences and PCR product length are listed in Supplemental Table S1.

### 2.5. Western Blot Analysis

Total protein extracts from liver were obtained by the Helenius method [22], and protein concentrations were determined by the Bradford method [23]. Thirty micrograms of protein extracts were subjected to SDS-polyacrylamide gel electrophoresis. The protein fractions were then transferred to Immobilon polyvinylidene difluoride transfer membranes (Millipore, Billerica, MA, USA), blocked for 1 h at room temperature with 5% non-fat milk solution in 0.1% Tween-20-Tris-Buffered Saline (TBST), and incubated as described previously [24]. Detection was performed using the ECL chemiluminescence kit for HRP (Amersham GE Healthcare Europe GmbH, Barcelona, Spain). To confirm the uniformity of protein loading, blots were incubated with β-tubulin antibody (Sigma-Aldrich, St. Louis, MO, USA) as a control. Primary antibodies for phosphorylated and total Akt and IκBα were supplied by Cell Signaling Technology (Danvers, MA, USA) and those for IRS-2 and phosphorylated and total IRβ were obtained from Santa Cruz Biotechnologies (Dallas, TX, USA). The antibody against phosphorylated IRS-2 was from Abcam (Cambridge, UK).

### 2.6. Statistical Analysis

The results are expressed as the mean of *n* values ± SEM. Plasma samples were assayed in duplicate. Significant differences between the values from the CT, F and W groups were established by one-way ANOVA and Šidák’s post-hoc test for selected comparisons; significant differences between the values from the W and WF groups were established by the unpaired *t*-test (GraphPad Software V6, La Jolla, CA, USA). When variance was not homogeneous, a non-parametric test was performed. The correlations between liver total cholesterol content and inflammation-related parameters were examined by two-tailed Pearson or Spearman Correlation analyses for data which follow a Gaussian distribution or not, respectively. The level of statistical significance was set at *p* ≤ 0.05.

## 3. Results

### 3.1. Western Diet ± Liquid Fructose Supplementation Does Not Trigger Visceral White Adipose Tissue Inflammation in LDL-R^−/−^ Mice

Zoometric parameters and liver lipid content in the animals used in the present study were previously reported [18], and are shown in Supplemental Table S2. Mice fed a Western-type diet (W group) showed an increase in body weight at the end of the study compared to controls (C), and the addition of liquid fructose (WF group) further increased final body weight. Importantly, liver and vWAT weights were only significantly increased in the WF group compared to the W group, despite the total amount of calories ingested by the mice from these groups were almost identical.

**Figure 1:**
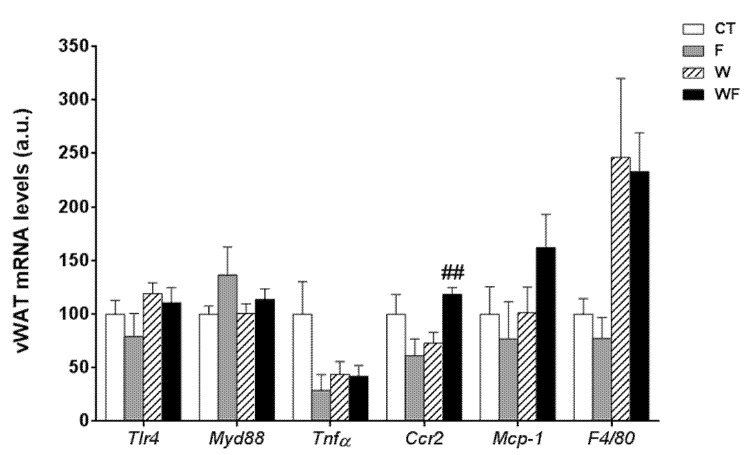
Bar plots showing the relative levels of specific mRNAs of pro-inflammatory molecules from Control (CT, mice fed standard solid-chow), Fructose (F, mice fed standard solid-chow plus a 15% fructose solution ad libitum), Western (W, mice fed Western solid-chow), and Western + Fructose (WF, mice fed Western solid-chow plus a 15% fructose solution ad libitum), represented as mean (a.u., arbitrary units) ± SEM of five different visceral adipose tissue samples. ## *p* < 0.01 vs. W values.

Using vWAT samples of LDL-R^−/−^ mice from the different dietary groups, we examined the expression of several pro-inflammatory molecules (Figure 1). The mRNA expression of toll-like receptor 4 (*tlr4*), which plays an essential role in the induction of pro-inflammatory cytokines in adipocytes, showed no differences among the groups. Similarly, the mRNA levels of the adaptor protein myeloid differentiation factor-88 (*myd88*) remained unaffected. In accordance with the lack of activation of the TLR4 pathway, the expression of the inflammatory cytokine tumour necrosis factor α (*tnf*α) was not significantly altered in vWAT in any of the dietary groups. C-C chemokine receptor type 2 (*ccr2*), which is involved in monocyte chemotaxis, showed a significant increase in the WF group compared to the W group (*p *< 0.01). However, the mRNA levels of monocyte chemoattractant protein-1 (*mcp-1*) and macrophage marker *f4*/*80* tended to increase, but not significantly, in these groups.

### 3.2. A Western-Type Diet Induces Hepatic Inflammation and Liquid Fructose Supplementation Enhances This Effect

**Figure 2:**
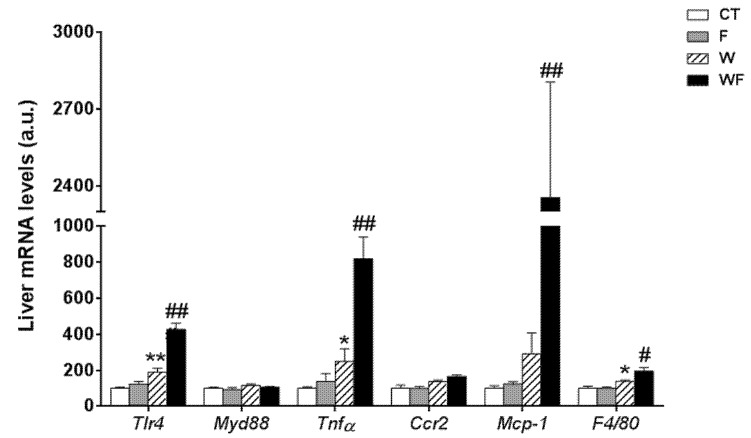
Bar plots showing the relative levels of specific mRNAs of pro-inflammatory molecules from Control (CT, mice fed standard solid-chow), Fructose (F, mice fed standard solid-chow plus a 15% fructose solution ad libitum), Western (W, mice fed Western solid-chow), and Western+Fructose (WF, mice fed Western solid-chow plus a 15% fructose solution ad libitum), represented as mean (a.u., arbitrary units) ± SEM of five different liver samples. * *p* < 0.05, ** *p* < 0.01 vs. CT values; # *p* < 0.05, ## *p* < 0.01 vs. W values.

In contrast to the lack of a clear inflammatory response in vWAT, the expression of most of the inflammatory markers determined in the liver was significantly increased by the Western-type diet alone and combined with liquid fructose supplementation (Figure 2). Thus, the expression of *tlr4* was induced by the Western diet (1.9-fold increase in W vs. CT group, *p *< 0.01), and supplementation with liquid fructose in this diet further enhanced the effect (2.3-fold increase in WF vs. W group, *p *< 0.01), although *myd88* mRNA levels remained unaffected. In accordance with *tlr4* induction, the mRNA level of the pro-inflammatory cytokine *tnfα* was increased in W compared to CT (2.5-fold, *p* < 0.05), and further induced after the Western diet was supplemented with fructose (3.3-fold increase in WF vs. W group, *p* < 0.01). Regarding the expression of genes related to monocyte recruitment and macrophage accumulation, our results showed no significant modification in the expression of *ccr2*, and a marked increase in the mRNA of *mcp-1* only in the WF group compared to W (8.1-fold, *p* < 0.01), whereas the expression of the macrophage marker *f4*/*80* was increased by the Western diet and further increased by fructose supplementation in this diet (1.4-fold, *p *< 0.05 W vs. CT and WF vs. W).

**Figure 3:**
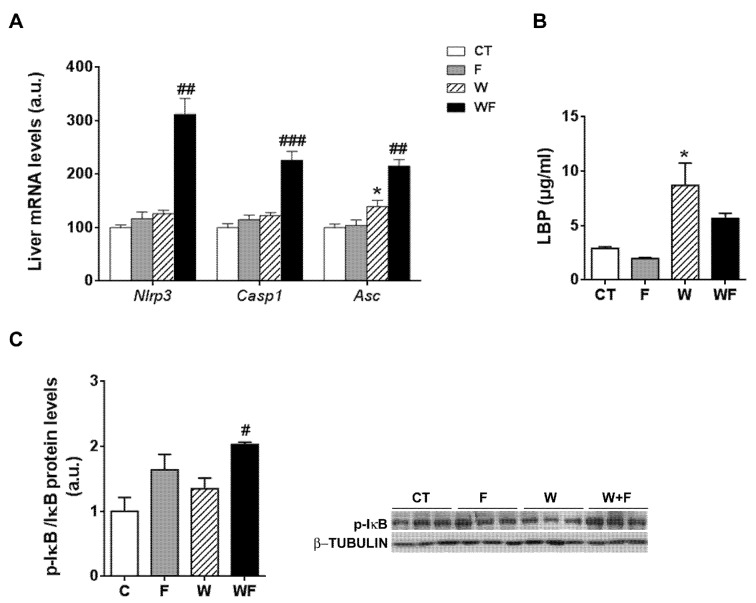
(**A**) Bar plots showing the relative levels of specific mRNAs of the NLRP3 inflammasome complex from Control (CT, mice fed standard solid-chow), Fructose (F, mice fed standard solid-chow plus a 15% fructose solution ad libitum), Western (W, mice fed Western solid-chow), and Western + Fructose (WF, mice fed Western solid-chow plus a 15% fructose solution ad libitum), represented as mean (a.u., arbitrary units) ± SEM of five different liver samples; (**B**) Plasma level of lipopolysaccharide (LPS)-binding protein represented as mean (μg/mL) ± SEM of five to six different plasma samples obtained from CT, F, W and WF groups; (**C**) Western-blot of phosphorylated and total IκBα in liver samples obtained from CT, F, W and WF groups. Representative bands corresponding to three different mice in each group are shown; bar plots show the level of the phosphorylated protein expressed as the mean (a.u., arbitrary units) ± SEM of the values obtained from three to four animals. * *p* < 0.05 vs. CT values; # *p* < 0.05, ## *p* < 0.01, ### *p* <0.001 vs. W values.

Given the inflammatory response observed in the liver, we assessed the involvement of the inflammasome in this process. To this end, the mRNA levels of several components of the nucleotide-binding domain, leucine-rich-containing family, pyrin domain-containing-3 (NLRP3) inflammasome were examined. As shown in Figure 3A, the mRNA expression of *nlrp3* and *caspase-1* were significantly higher in the WF group compared to the W group (2.5-fold, *p* < 0.01 and 1.8-fold, *p* <0.001, respectively). Similarly, the mRNA expression of the adaptor molecule *asc* (apoptosis-associated speck-like protein containing a caspase-recruitment domain) was increased by the Western-type diet (1.3-fold, *p* < 0.05) and further enhanced by the addition of fructose to this diet (1.5-fold increase in WF vs. W group, *p *< 0.01).

One of the pathways that initiates inflammasome activation is mediated by microbial signals, such as lipopolysaccharide (LPS), which upregulate NLRP3 expression through nuclear factor κB (NFκB) [25]. Plasma level of LPS-binding protein (LBP), a biomarker of circulating LPS, was unaffected by fructose supplementation, but increased in the W group compared to the CT group (3-fold, *p *< 0.05) (Figure 3B). To determine NFκB activity, we measured the amount of phosphorylated inhibitor of κB (p-IκB), as the classical pathway of NFκB activation involves IκB phosphorylation and degradation, which favors NF-κB nuclear translocation and transcriptional activity. Our results show that p-IkB levels were increased significantly in the WF group compared to W (Figure 3C).

### 3.3. Addition of a Liquid Fructose Supplement to a Western-Type Diet Increases Fibrogenesis Markers in the Liver of LDL-R^−/−^ Mice

**Figure 4:**
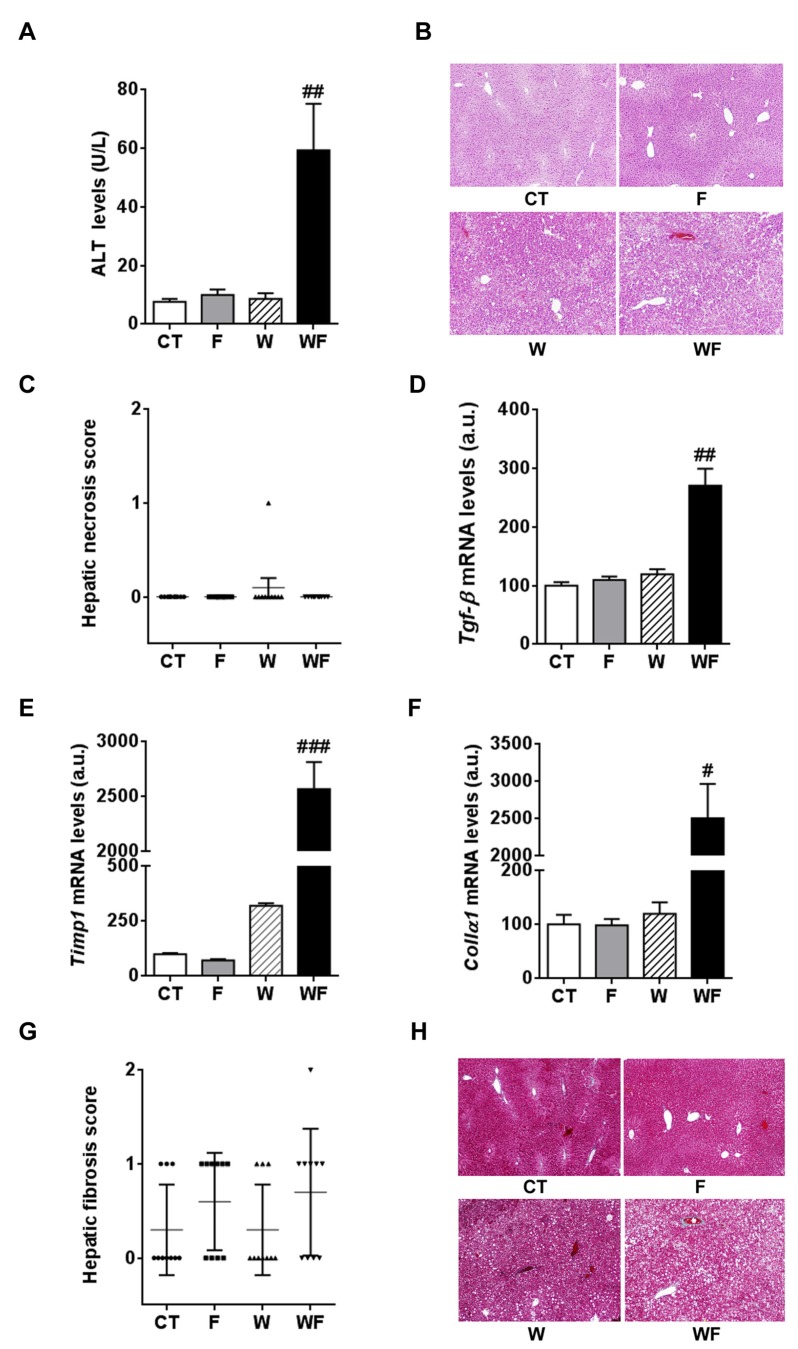
(**A**) Alanine aminotransferase (ALT) activity expressed as mean (U/L) ± SEM of five to six different plasma samples from Control (CT, mice fed standard solid-chow), Fructose (F, mice fed standard solid-chow plus a 15% fructose solution ad libitum), Western (W, mice fed Western solid-chow), and Western + Fructose (WF, mice fed Western solid-chow plus a 15% fructose solution ad libitum); (**B**,**C**) Histological examination of necrosis by hematoxylin and eosin staining. A representative image of liver sections from each experimental group is shown (magnification 10×). The scatter plot shows individual values of necrosis degree (0, absent; 1, <1%). The horizontal line across each group locates the mean, and bars indicate the SEM; (**D**–**F**) Bar plots showing the relative levels of specific mRNAs of fibrogenesis-related molecules, represented as mean (a.u., arbitrary units) ± SEM of five different liver samples obtained from CT, F, W and WF groups. # *p* < 0.05, ## *p* < 0.01, ### *p* < 0.001 vs. W values; (**G**,**H**) Histological examination of fibrosis by Masson’s trichrome acid staining. The scatter plot shows individual values of fibrosis score (0, no fibrosis; 1, portal or sinusoidal fibrosis without septa; 2, portal or sinusoidal fibrosis with rare septa). The horizontal line across each group locates the mean, and bars indicate the SEM. A representative image of liver sections from each experimental group is shown (magnification 10×).

Plasma ALT activity, which is commonly used to monitor liver injury, showed a marked increase in the WF group compared to the W group (6.8-fold, *p *< 0.01, Figure 4A), although hematoxylin and eosin staining did not reveal apparent necrosis in any of the groups (Figure 4B,C).

Then, we measured the expression of factors related to fibrogenesis key events such as stellate cell activation and matrix deposition. The hepatic mRNA levels of transforming growth factor β (*tgf-β*) and tissue inhibitor of metalloproteinase 1 (*timp-1*) were markedly and significantly increased only in the WF group, compared to the W group (Figure 4D,E). Consistent with this induction, hepatic collagen α1 (*collα1*) mRNA levels were markedly increased only in the WF mice (Figure 4F). We then assessed the degree of hepatic fibrosis by examining the Masson’s trichrome-stained liver sections. As shown in Figure 4G,H, none of the diets caused overt liver fibrosis; the semi-quantitative analysis of fibrosis scores indicated that only in the fructose-supplemented groups there were 50%–70% of mice with score 1, and in the WF group there was one animal which reached grade 2.

### 3.4. Fructose Supplementation and a Western Diet Reduce Basal Insulin Receptor Substrate-2 (IRS-2) Expression but Do Not Impair Insulin-Stimulated Akt Phosphorylation in the Liver of LDL-R^−/−^ Mice

**Figure 5:**
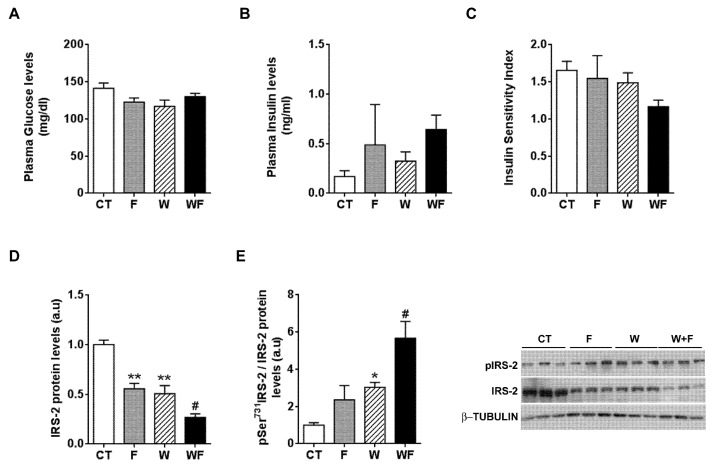
Insulin receptor signalling under basal conditions. Glucose (**A**) and insulin (**B**) plasma levels from Control (CT, mice fed standard solid-chow), Fructose (F, mice fed standard solid-chow plus a 15% fructose solution ad libitum), Western (W, mice fed Western solid-chow), and Western + Fructose (WF, mice fed Western solid-chow plus a 15% fructose solution ad libitum), expressed as mean ± SEM of 9–10 different samples; (**C**) Insulin Sensitivity Index (ISI), calculated as [2/(blood insulin (nM) × blood glucose (µM) + 1]; Western-blot of total and phosphorylated IRS-2 (**D**,**E**) proteins in liver samples obtained from the different experimental groups under basal conditions. Representative bands corresponding to three different mice in each group are shown; bar plots show the level of total IRS-2 expressed as the mean (a.u., arbitrary units) ± SEM of the values obtained from three to four animals, and the ratio between phosphorylated and total IRS-2. * *p *< 0.05 and ** *p* < 0.01 vs. CT values; # *p* < 0.05 vs. W values.

Plasma glucose and plasma insulin levels remained essentially unaffected by the different dietary interventions (Figure 5A,B). When we calculated the insulin sensitivity index (ISI), we observed a non-significant trend to reduction in the WF group (Figure 5C). In addition, we calculated another surrogate index of insulin sensitivity, the HOMA-IR, and the results also pointed to lower insulin sensitivity, especially in the WF group compared to W, although it did not reach statistical significance (1.7 ± 0.6, 4.3 ± 3.6, 3.0 ± 1.1 and 5.9 ± 1.2, expressed as mean ± SEM, for C, F, W and WF groups). Thus, we wondered whether the establishment of an inflammatory and pro-fibrotic status in the liver could alter insulin signalling in this tissue. The basal expression of IRS-2, one of the main transducers of the insulin signal in the liver, was reduced in F and W groups vs. CT, and further diminished in WF vs. W (Figure 5D). We also determined the amount of IRS-2 phosphorylated at Ser731, as phosphorylation at this position has been related to impaired insulin signal transduction [26]. Our results showed significant increases in IRS-2 phosphorylation status in the W group compared to the CT group and in the WF group versus the W group (Figure 5E).

**Figure 6:**
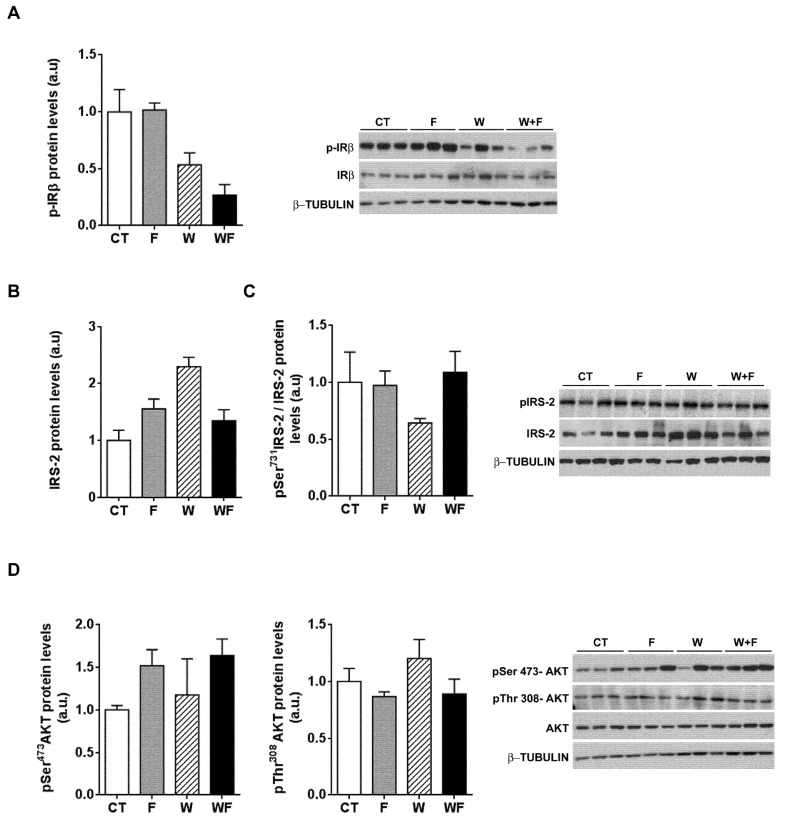
Insulin receptor signalling after an acute insulin challenge. Western-blot of total and phosphorylated IR-β (**A**), IRS-2 (**B**,**C**) and Akt (**D**) proteins in liver samples obtained from the different experimental groups, after exogenous insulin administration (0.15 units/g body weight). Representative bands corresponding to three different mice in each group are shown; bar plots show the level of the proteins expressed as the mean (a.u., arbitrary units) ± SEM of the values obtained from three to four animals. For IRS-2, as the corresponding total protein levels vary significantly between groups, the bar plot shows the ratio between phosphorylated and total protein levels.

To further explore whether hepatic insulin sensitivity was impaired, we injected insulin (0.15 units/g body weight) in a subset of rats and 15 minutes later we assessed the phosphorylation status of several molecules related to insulin signalling: the beta subunit of the insulin receptor (IR-β), IRS-2 and Akt. As shown in Figure 6A, insulin-induced IR-β phosphorylation tended to be lower in W and WF groups, but these differences did not reach significance. On the other hand, under acute insulin-stimulated conditions, neither the phosphorylation of IRS-2 at Ser731, nor Akt phosphorylation at both Thr308 and Ser473 positions were modified by any of the dietary interventions (Figure 6B–D).

## 4. Discussion

Here we show that consumption of a Western-type diet for 12 weeks causes an inflammatory reaction in the liver of male LDL-R^−/−^ mice that is exacerbated by 15% *w*/*v* liquid fructose supplementation, which also increases fibrogenesis markers. However, vWAT is not inflamed and hepatic insulin-induced Akt phosphorylation is not hampered, despite a clear reduction in basal IRS-2 levels.

In this study we used LDL-R^−/−^ mice, a model of diet-induced atherosclerosis that is also suitable for investigating the progress from hepatic steatosis to non-alcoholic steatohepatitis (NASH) [19]. We fed these mice either a standard rodent chow diet or a Western-type diet rich in cholesterol (0.21%), saturated fat (21%) and sucrose (34%) to provide a high fructose intake [18]. A high level of fructose in the diet is required to mimic all human NASH features in rodents and is typical of the diet consumed by humans with NASH [27]. Moreover, we added liquid fructose to both diets (15% *w*/*v*), since unhealthy human diets are also characterised by a high intake of beverages sweetened with sucrose or high-fructose corn syrup (both of which contain fructose), and this has been linked to the acquisition of NAFLD and its progression to NASH and hepatic fibrosis [28,29].

**Figure 7:**
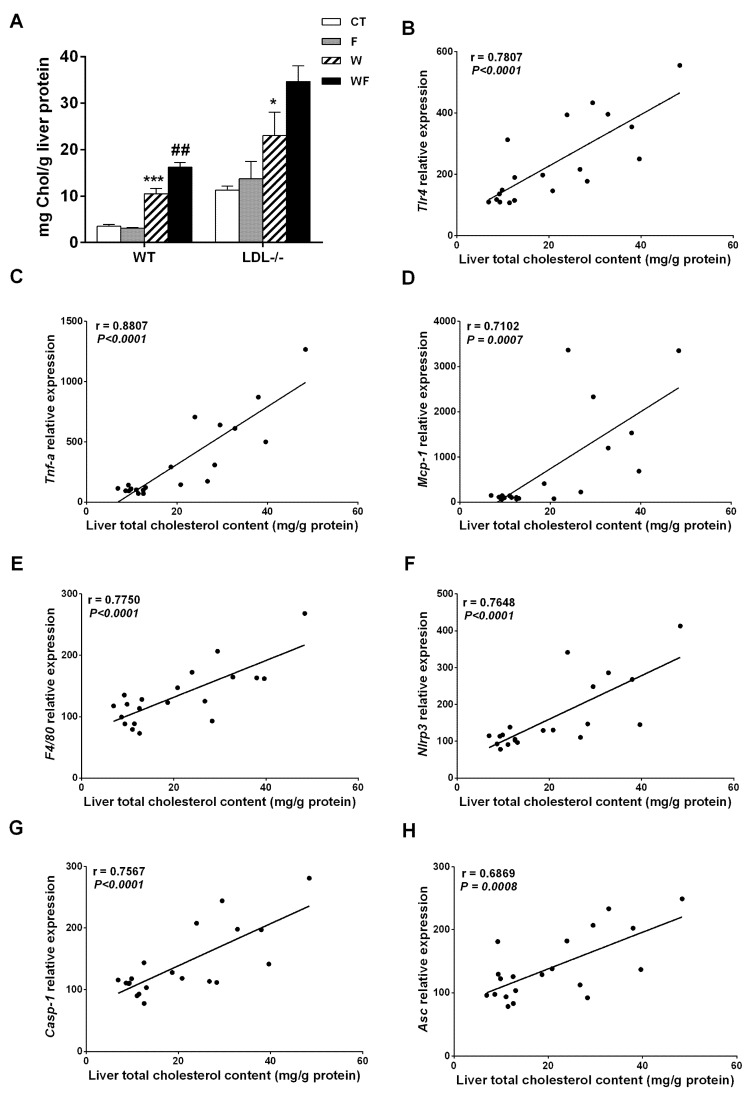
(**A**) Liver cholesterol levels from wild-type (WT) and LDL-R^−/−^ mice fed standard solid-chow (CT), standard solid-chow plus a 15% fructose solution ad libitum (F), Western solid-chow (W), and Western solid-chow plus a 15% fructose solution ad libitum (WF), expressed as mean ± SEM of 9–10 animals; Data were obtained from [17,18]. Correlation between liver cholesterol content and mRNA expression of pro-inflammatory molecules including: (**B**) toll-like receptor 4 (*tlr4*); (**C**) tumor necrosis factor (*TNF-α*); (**D**) monocyte chemoattractant protein-1 (*mcp-1*); (**E**) f4/80; (**F**) NLR family pyrin domain containing 3 (*nlrp3*); (**G**) caspase-1 (*casp1*) and (**H**) apoptosis-associated speck-like protein containing a caspase-recruitment domain (*asc*). Statistical analysis was performed using two tailed Pearson or Spearman correlation. R indicates correlation coefficient.

Bieghs et al. [19] showed that LDL-R^−/−^ mice develop sustained hepatic inflammation upon high cholesterol feeding due to the uptake of oxidized low-density lipoproteins through the CD36 receptor. Moreover, it has been shown that hepatic cholesterol accumulation is one of the main drivers of hepatic inflammation in LDL-R^−/−^ mice [30]. In the present study, only mice with the highest liver cholesterol content (W and WF groups) show a clear inflammatory response in this tissue, and the response is more intense in the WF group (Supplemental Table S2 and Figure 2). This is in agreement with our previous observation of increased CD36 mRNA expression in WF-LDL-R^−/−^ mice [18]. In contrast, our recent study performed in wild type C57BL/6N mice, showed no signs of hepatic inflammation despite a significant increase in hepatic cholesterol deposition in W and WF groups [17]. To compare the hepatic cholesterol deposition between wild type and knockout mice, we used data from [17,18], and expressed them as mg of cholesterol per gram protein. This is more accurate for comparison, due to the significant increase in liver weight observed in LDL-R^−/−^ mice from the WF group, but not in wild-type mice. As shown in Figure 7A, the amount of cholesterol per gram of liver protein in control LDL-R^−/−^ mice is already as high as the amount observed in wild type mice fed with the Western-type diet, and W and WF LDL-R^−/−^ mice more than doubled the hepatic cholesterol compared to the same dietary groups in wild type mice. The presence of hepatic inflammation in LDL-R^−/−^ but not in wild type mice may thus be related to the larger cholesterol accumulation in the liver of knockout mice even when fed a healthy control diet. In support of this hypothesis, we found significant correlations between total cholesterol content in the livers of LDL-R^−/−^ mice and the hepatic expression of the inflammatory markers *tlr4*, *tnfα*, *mcp-1*, *f4*/*80*, as well as the inflammasome components *nlrp3*, *caspase-1* and *asc* (Figure 7B–H).

However, other pathways besides cholesterol accumulation may be involved in the onset of hepatic inflammation and its exacerbation by fructose addition. It has been reported that liquid fructose supplementation (30% *w*/*v*) in mice increases intestinal permeability and thus allows bacterial endotoxins to enter portal blood, which then leads to the development of liver TLR4-mediated inflammation [31,32]. Our results show that liquid fructose supplementation in the LDL-R^−/−^ model does not seem to increase blood endotoxins, as revealed by unchanged plasma levels of LBP, a biomarker of circulating lipopolysaccharide (Figure 3B). This lack of effect can be attributed to the use of a different strain or the lower concentration of fructose solution used in our study (15 vs. 30% *w*/*v*). However, we show here that plasma LBP increases significantly in mice fed a Western-type diet compared to the chow-fed mice (Figure 3B). This suggests that the increase in intestinal permeability to bacterial toxins in our model may be caused by some component of the Western diet. Thus in WF mice, which ingest a smaller amount of solid food than the W group [18], the amount of plasma LBP is also somewhat reduced, albeit not significantly (Figure 3B). Gut-derived bacterial components act as a first signal to prime the inflammasome, through binding to TLRs and subsequent NFκB activation leading to the upregulation of NLRP3 transcription [25]. In accordance with the higher LBP levels, our results show that the Western-type diet increases *tlr4* mRNA expression and that fructose supplementation enhances this effect. However, significant NFκB activation (Figure 3C) and *nlrp3* induction (Figure 3A) are only observed in the WF group, suggesting that fructose or the Western-type diet alone do not suffice to fully prime the inflammasome. Activation of the TLR4-NFκB pathway enables a second, non-microbial-derived signal to activate the NLRP3 inflammasome [25]. It has been shown that cholesterol crystals activate the NLRP3 inflammasome [33], and as we have mentioned before the highest hepatic cholesterol levels are only achieved when fructose is added to a Western-type diet. Thus, it is plausible to suggest that fructose added to a healthy dietary substrate is not potent enough to activate the inflammasome, but it may act as a second step contributing to full inflammasome activation in the WF group.

Inflammasome activation leads to the induction of *mcp-1* and thus to the recruitment of inflammatory cells into the damaged tissue [25]. Accordingly, although *mcp-1* and *f4*/*80* mRNA levels are gradually increased by the dietary interventions, and the increase is maximal in the group in which inflammasome is fully activated, i.e., the WF group (Figure 2). Inflammasome activation also stimulates stellate hepatic cells through increased IL-1 secretion and leads to fibrosis [25]. This is also consistent with our results, since the expression of the critical inducers of fibrogenesis (*tgfβ* and *timp-1*), as well as the expression of one of the predominant collagens in the fibrotic liver (*collα1*), are only significantly increased in livers from the WF group (Figure 4D–F). Liver fibrosis is a dynamic process, where progression to advanced fibrotic stages is characterized by increased synthesis and decreased degradation of extracellular matrix (ECM) proteins, whereas the opposite may lead to regression or reversal [34,35]. Several studies in humans and animal models suggest a prominent role for TIMP-1 in this process, as TIMP-1 up-regulation by inflammatory cytokines, especially TGFβ, shifts the balance toward ECM synthesis and fibrogenesis [35,36]. TIMP-1 not only prevents the degradation of the accumulated ECM by blocking the function of matrix metalloproteases, thus opposing to fibrosis reversal, but also inhibits the apoptosis of activated hepatic stellate cells, the main cellular source of type I collagen [37]. Thus, although histological signs of overt hepatic fibrosis are still absent in our model (Figure 4G,H), the marked induction of TIMP-1 in the WF group suggest that with longer exposure to this WF diet fibrosis would continue unopposed, leading to progression to advanced fibrotic stages.

The presence of hepatic steatosis [18] and the inflammatory and pro-fibrogenic status in the liver of LDL-R^−/−^ mice caused by the combined effects of fructose and the Western diet prompted us to examine the effects of the dietary interventions on the hepatic insulin signalling cascade. Although plasma glucose and insulin levels do not vary significantly between groups, whole-body insulin sensitivity (measured by the ISI) tends to decrease in the WF group. Moreover, under basal conditions, the amount of total IRS-2 is reduced and phosphorylation of IRS-2 at Ser731 is increased across the different dietary groups, which suggest an impairment of hepatic insulin receptor signalling cascade (Figure 5). However, after an insulin challenge total IRS-2 expression is not reduced and its phosphorylation status does not vary significantly. In addition, the phosphorylation of Akt at Thr308 and Ser473, which lead to full Akt activation after insulin stimulation [38,39,40], is not impaired by any of the dietary regimes (Figure 6). These results suggest that despite there is some degree of impairment in insulin receptor signalling under basal conditions, when insulin levels are high this deficit is overcome and signal transduction proceeds correctly.

Our results showing inflammation and signs of progression to fibrosis contrasts with the prevalent theory that links these phenomena with IR [2,3,4,5]. However, it has been suggested that insulin sensitivity is not reduced when the inflammation is restricted to the liver compartment and does not affect adipose tissue [1,11,41]. Our results are in accordance with this hypothesis, as LDL-R^−/−^ mice show no signs of inflammation in vWAT (Figure 1), whereas in our previous study in wild type mice we observed inflammation in vWAT but not in the liver, and whole-body insulin sensitivity was significantly reduced [17].

In contrast to our results, Subramanian et al. [30] found a marked inflammatory reaction in epididymal adipose tissue from LDL-R^−/−^ mice that were fed a diabetogenic diet with 0.15% cholesterol, which was linked to whole-body IR. Although the amount of cholesterol in the Western-type diet was even greater in our study (0.21%), we used a shorter feeding period (12 weeks instead of 24 weeks). Long-term studies (5.5 months) also show that a Western-type diet similar to the one used in the present study causes IR with hyperglycaemia and hyperinsulinemia [42]. Therefore, we cannot rule out the possibility that longer exposure to the Western-type diet, with or without liquid fructose supplementation, could also induce adipose tissue inflammation and IR. However, in a recent study with a relatively short duration (16–20 weeks), LDL-R^−/−^ mice fed diets that provided more calories from fat (58%–60%) and fewer calories from carbohydrate (20%–28%) than our Western-type diet (40 kcal% fat and 43 kcal% carbohydrate) showed inflammation in both liver and adipose tissue, accompanied by IR and impaired glucose tolerance [43]. Therefore, both the duration of the study and the type of nutrients provided by the diet determine the metabolic response.

Taken together, our results suggest that liquid fructose supplementation in a Western-type diet in LDL-R^−/−^ mice aggravates the inflammatory response in the liver and initiates fibrogenesis, but the lack of inflammation in vWAT probably delays the manifestation of glucose intolerance and impaired hepatic insulin signalling.

## Supplementary Materials

The following are available online at http://www.mdpi.com/2072-6643/9/3/278/s1, Table S1: Primers used for RT-PCR., Table S2: Zoometric parameters of LDLR^−/−^ mice exposed to four different dietary regimes.
