# Supplementary material for: The Addition of Liquid Fructose to a Western-Type Diet in LDL-R−/− Mice Induces Liver Inflammation and Fibrogenesis Markers without Disrupting Insulin Receptor Signalling after an Insulin Challenge
Source: Nutrients. 2017 Mar 15;9(3):278. doi: 10.3390/nu9030278 (PMC5372941; doi:10.3390/nu9030278)
Supplement: Supplementary file 1 [file nutrients-09-00278-s001.docx]

The Addition of Liquid Fructose to a Western-Type Diet in LDL-R^−/−^ Mice Induces Liver Inflammation and Fibrogenesis Markers without Disrupting Insulin Receptor Signalling after an Insulin Challenge

Gemma Sangüesa, Miguel Baena, Natalia Hutter, José Carlos Montañés,
Rosa María Sánchez, Núria Roglans, Juan Carlos Laguna and Marta Alegret

**Table S1.** Primers used for RT-PCR.

| **Gen** | **GenBankTM nº** | **Primer sequences** | **PCR product** |
| --- | --- | --- | --- |
|  |  |  |  |
| *Asc* | NM_023258.4 | Forward: 5'-TGCAACTGCGAGAAGGCTAT-3' | 117 bp |
|  |  | Reverse: 5'- TGAGCTCCAAGCCATACGAC-3' |  |
| *Casp1* | NM_009807.2 | Forward: 5'-CTGGGACCCTCAAGTTTTGC -3' | 103 bp |
|  |  | Reverse: 5'- AGACGTGTACGAGTGGTTGT-3' |  |
| *Ccr2* | NM_009915.2 | Forward: 5'-AGAGGTCTCGGTTGGGTTGT-3' | 100 bp |
|  |  | Reverse: 5'- CACTGTCTTTGAGGCTTGTTGC-3' |  |
| *Colα1* | NM_008607.2 | Forward: 5'- GACGTTCAAGGAATTCAGTTTCTTT-3' | 72 bp |
|  |  | Reverse: 5'- TGGTGTTTTGGGATGCTTAGG-3' |  |
| *F4/80* | XM_006523601.1 | Forward: 5'- GGAGGACTTCTCCAAGCCTATT-3' | 69 bp |
|  |  | Reverse: 5'- GGCCTCTCAGACTTCTGCTTT -3' |  |
| *Mcp-1* | NM_011333.3 | Forward: 5'-GCTGGAGAGCTACAAGAGGATCA-3' | 79 bp |
|  |  | Reverse: 5'- CTCTCTCTTGAGCTTGGTGACAAA-3' |  |
| *Myd88* | NM_010851.2 | Forward: 5'- AGGCGATGAAGAAGGACTTTCC-3' | 163 bp |
|  |  | Reverse: 5'- TCAGTCTCATCTTCCCCTCTGC-3' |  |
| *Nlrp3* | NM_145827.3 | Forward: 5'- TGACCCAAACCCACCAGTGT -3' | 71 bp |
|  |  | Reverse: 5'- CAAAGCCATCCATGAGGAAGA -3' |  |
| *Tbp* | NM_013684.3 | Forward: 5’-TGCCACACCAGCTTCTGAGA-3’ | 79 bp |
|  |  | Reverse: 5’-TTTACAGCCAAGATTCACGGTAGA-3’ |  |
| *Tgfβ* | NM_011577.2 | Forward: 5’- GACTCTCCACCTGCAAGACC -3’ | 100 bp |
|  |  | Reverse: 5’- GGACTGGCGAGCCTTAGTTT -3’ |  |
| *Timp1* | NM_001044384.1 | Forward: 5’- CCAGAACCGCAGTGAAGAGT -3’ | 93 bp |
|  |  | Reverse: 5’- GTACGCCAGGGAACCAAGAA -3' |  |
| *Tlr4* | NM_021297.3 | Forward: 5'- GGCTCCTGGCTAGGACTCTGA-3' | 114 bp |
|  |  | Reverse: 5'- TCTGATCCATGCATTGGTAGGT-3' |  |
| *Tnfα* | NM_013693.3 | Forward: 5’-GAAAAGCAAGCAGCCAACCA -3’ | 106 bp |
|  |  | Reverse: 5’-CGGATCATGCTTTCTGTGCTC-3’ |  |

*Asc:* apoptosis-associated speck-like protein containing a caspase-recruitment domain; *Casp-1*: caspase-1; *Ccr2:* C-C chemokine receptor type 2; *Collα1:* collagen α1; *Mcp-1:* monocyte chemoattractant protein-1; *Myd88:* myeloid differentiation factor-88; *Nlrp3*: nucleotide-binding domain, leucine-rich-containing family, pyrin domain-containing-3; *Tbp*: TATA box-binding protein; *Tgf-β:* transforming growth factor β; *Timp-1:* tissue inhibitor of metalloproteinase 1; *Tlr4:* toll-like receptor 4; *Tnf*α: tumour necrosis factor α.

**Table S2.** Zoometric parameters of LDLR^−/−^ mice exposed to four different dietary regimes.

| **Parameters** | **CT** | **F** | **W** | **WF** |
| --- | --- | --- | --- | --- |
| Final body weight (g) | 26. 6 ± 0.8 | 26.5 ± 1.6 | 33.0 ± 1.9* | 39.2 ± 2.7^#^ |
| Liver weight (g) | 1.38 ± 0.10 | 1.37 ± 0.06 | 1.60 ± 0.09 | 2.35 ± 0.27^##^ |
| % Liver/body weight | 4.9 ± 0.4 | 5.3 ± 0.9 | 4.9 ± 0.6 | 6.1 ± 1.2 |
| vWAT weight (g) | 0.88 ± 0.15 | 1.08 ± 0.16 | 1.60 ± 0.46 | 3.25 ± 0.16^##^ |
| % vWAT/body weight | 3.1 ± 1.1 | 3.7 ± 1.0 | 4.2 ± 2.3 | 8.0 ± 0.6^#^ |
| Total ingested kcal (animal × 84 days) | 840 ± 9 | 946 ± 14 | 1252 ± 125* | 1262 ± 8 |
| Liver cholesterol content (mg/g liver protein) | 11.0 ± 0.8 | 13.7 ± 3.8 | 23.0 ± 5.0* | 34.6 ± 0.8 |

CT: Standard solid-chow; F: Standard solid-chow plus a 15% fructose solution ad libitum; W: Western solid-chow; WF: Western solid-chow plus a 15% fructose solution ad libitum; vWAT: Visceral White Adipose Tissue. Values are expressed as mean ± SEM of 5–10 animals except for total ingested kcal, which were obtained from two cages containing at least 4 animals each. * *p* < 0.05 vs C; ^#^ *p* < 0.05 vs W; ^##^ *p* < 0.01 vs W.
